# Health Equity in All Policies: Recommendation from the European Actions

**DOI:** 10.7189/jogh.14.03002

**Published:** 2024-08-09

**Authors:** Raffaella Bucciardini, Tuulia Rotko, Anna Maria Giammarioli

**Affiliations:** 1 National Center for Global Health, Istituto Superiore di Sanità, Rome, Italy; 2 Finnish Institute for Health and Welfare, Helsinki, Finland

Health inequalities are determined only in part by the health system; most are caused by the actions of non-health sectors or their policies, such as education, employment, environment, agriculture, or immigration, among others. People's health and well-being are determined by the circumstances and conditions of their daily life, which are, in turn, influenced by factors and policies that primarily emerge from non-health sectors [1,2]. This makes it clear that only the collaboration and integration of different policy sectors that share the same objective of ‘equity’ can allow each policy to become responsible for the portion of action of its competence and all together contribute to significant results in terms of impact on inequalities. It is indeed in this context that the Health Equity in All Policies (HEiAP) approach is introduced, which stresses how a whole-of-government approach integrating both the actions of the health and non-health sectors can more significantly impact population health and health equity [3–5]. The issue of health inequalities and the HEiAP approach has not yet systematically entered the agenda of European or global government. Awareness and attention to these issues, and especially their application in the field, significantly differ between and within countries [6–8].

## EFFORT TO ADDRESS HEIAP AT THE EUROPEAN LEVEL

At European level, this topic was significantly addressed through the Joint Action Equity Action (2011–14) included in the Second Programme of Community action in the Field of Health, 2008–13 [9], which identified opportunities and barriers for the implementation of the HEiAP approach in Europe. The final Equity Action conference took place on 23 January 2014 in Brussels and was attended by international health inequality experts, officials, and policy makers from EU Member States, the EU itself, and a range of key stakeholders [10].

Further, a new Joint Action (Joint Action Health Equity Europe) was initiated in 2018 with the aim of fostering a cooperative approach among EU countries and implementing concrete actions to reduce health inequalities in key settings, such as those related to strengthening capacity and commitment in all sectors across governance at national, regional and local level. Fourteen European countries (Belgium, Bulgaria, Croatia, Finland, Greece, Italy, Lithuania, Netherlands, Poland, Portugal, Romania, Slovenia, Spain, and the United Kingdom) implemented concrete policy actions to improve governance concerning the HEiAP approach [11]. Based on their results, four recommendations were produced on how to promote health equity in the contest of Health in All Policies and governance.

## RECOMMENDATIONS

### Use data and evidence to increase understanding about health inequality, health equity, and social determinants of health

This first recommendation highlights a need for the availability of quantitative and qualitative data at the national, regional, and local level, as well as from vulnerable groups and communities, and different sectors. It also stresses a need to establish links between otherwise disparate sectors in this regard. Further, analysing regional/local comparable data seems to be the first step to recognising health inequalities in a country. A minimum data set of health inequalities indicators is found to be necessary for the purpose of HEiAP. Lastly, this recommendation suggests establishing a national multidisciplinary platform (integrating data from different sectors) for assessing the impact of policy measures on health inequalities, and focussing on equity when assessing the impact of policy measures.

### Cross-sectoral work is necessary to strengthen commitment, build mutual trust and set common goals

Building partnerships with different sectors and organisations to gain know/how and future collaboration emerges as an important topic within the second recommendation. In this context, both horizontal (cross-sectoral) and vertical (national-regional-local including citizens/communities) collaboration us needed, with non-governmental organisations being a powerful tool that need to be involved.

The recommendation also puts forward the necessity of involving policy actors. Political commitment is key to support this process, with an important note that the political actors involved are more receptive during electoral campaign. In this framework, a link to authorities and policymakers should also be established. Specifically, all kinds of authorities could be put to work together, whereby a responsible party could be set together to facilitate the work. Health sector should take at least an advocacy role in this process.

### Build capacity on health inequality and make it practical

This recommendation covers a regular mapping of the capacity and needs of entities/authorities responsible for planning and implementation of national/regional/local public policies having impact on health. This means proposing policies and measures that reduce health inequality and providing concrete tools (e.g. health inequality assessments, checklists) that different sectors can take to reduce inequality; integrating them to existing work by working interconnected with other existing policies and integrating a focus on equity into the required procedures; and reviewing/establishing new governance mechanisms (if needed) in public health at national, regional, and local levels.

**Figure:**
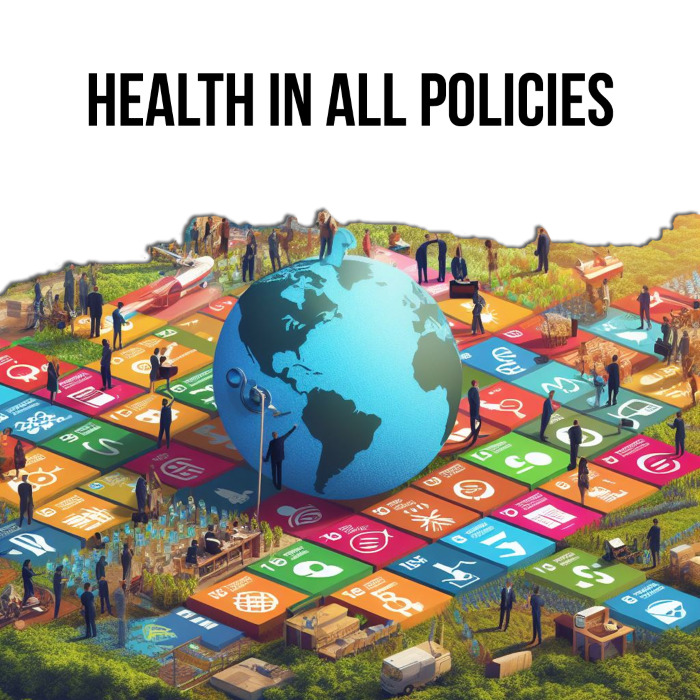
Photo: HEiAP proposes a multifaceted approach to address the social determinants of health that influence people's health and well-being. These determinants include the ‘causes of the causes’ of health inequalities, referring to the unequal conditions in which individuals are born, grow up, live and work. Source: created and provided by authors.

### Be prepared to use windows of opportunities and recognise strategic opportunities for reducing health inequality

This recommendation notes the importance of scanning and recognising windows of opportunities; for example, the coronavirus disease 2019 (COVID-19) pandemic forced us all to put public health in the centre of all policies. While it was an exceptional opportunity, there is now a need to enlarge the view for other health and social policies. Lastly, stakeholders should be ready to react/use windows of opportunities when available and recognise strategic opportunities by linking health equity and health inequality with other developments such as climate issues or poverty, highlighting such links, create bonds between them and working jointly between sectors.

## CONCLUSIONS

Improving population health and health equity normally takes much longer than most government tenures, making time frames and sustainability particularly difficult for HEiAP [12,13].

Robust evidence has shown that coordinated policy actions on the social determinants of health can have several effects, such as reducing health gaps, improving the overall health of the entire population, and promoting substantial returns and positive impact on the national economic performance [14].

In this regard, consistent structures are needed for a broad-based collaboration to accomplish common trust, commitment, and accountability, within which objectives should be formulated jointly with responsibilities shared among the various sectors. If inequalities are reduced in all sectors (employment, education, environment, etc.), this will lead to a more equal and desired health outcomes in the long-term. It is imperative to consider all citizens and their different needs and adjust actions/measures accordingly (e.g. proportional universalism).
